# Supplementary material for: Personality and emotional intelligence of researchers: The importance of affects
Source: PLoS One. 2024 Aug 9;19(8):e0304905. doi: 10.1371/journal.pone.0304905 (PMC11315323; doi:10.1371/journal.pone.0304905)
Supplement: Attachment — Submitted filename: Response_to_reviewers.docx [file pone.0304905.s001.docx]

June 9th, 2023

Dear Professor Sohaib Mustafa,

Thank you for your invitation to revise and resubmit our paper and for giving us detailed instructions about how to perform the revision.

We appreciate your comments very much as well as the comments from two reviewers. All comments have provided extremely valuable feedback, which has helped us improve our paper.

With regards to your comments, we have provided detailed responses for each one:

***Comment #1***

Please ensure that your manuscript meets PLOS ONE's style requirements, including those for file naming. The PLOS ONE style templates can be found at

[https://journals.plos.org/plosone/s/file?id=wjVg/PLOSOne_formatting_sample_main_body.pdf](https://urldefense.com/v3/__https:/journals.plos.org/plosone/s/file?id=wjVg*PLOSOne_formatting_sample_main_body.pdf__;Lw!!D9dNQwwGXtA!U30fcoaDEH2rdboyNKiMbIF3z_XqAMtd7GioIokmddrM0o1DPXAhSRmJROJIeTuwMB5RH1grUPGBnCIwjClg$) and

[https://journals.plos.org/plosone/s/file?id=ba62/PLOSOne_formatting_sample_title_authors_affiliations.pdf](https://urldefense.com/v3/__https:/journals.plos.org/plosone/s/file?id=ba62*PLOSOne_formatting_sample_title_authors_affiliations.pdf__;Lw!!D9dNQwwGXtA!U30fcoaDEH2rdboyNKiMbIF3z_XqAMtd7GioIokmddrM0o1DPXAhSRmJROJIeTuwMB5RH1grUPGBnK9LFkuY$)

Response: Thank you for reminding us of this issue. After consulting both files, we consider that this article meets all PLOS ONE style requirements.

***Comment #2***

Please provide additional details regarding ethical approval in the body of your manuscript. In the Methods section, please ensure that you have specified the name of the IRB/ethics committee that approved your study.

Response: We included the following statement in the Methods section: “The project received a favourable report from the Ethics Committee of the Spanish National Research Council (CSIC) and from the Polytechnic University of Valencia”.

***Comment #3***

Please include your full ethics statement in the ‘Methods’ section of your manuscript file. In your statement, please include the full name of the IRB or ethics committee who approved or waived your study, as well as whether or not you obtained informed written or verbal consent. If consent was waived for your study, please include this information in your statement as well.

Response: You are totally right. In fact, we passed an evaluation of the study before conducting it and we included all the feedback in the performance of our study in order to reach proficiency in Ethical procedure. Thanks to your comment we have inserted a sentence along these lines, including a mention to the granting ethical committees.

***Comment #4***

We noted in your submission details that a portion of your manuscript may have been presented or published elsewhere. "An earlier version of this paper was presented at the V National Congress of Psychology and International Symposium on Public Health Psychology (CNP2021), Online, July 9-11, 2021. A preprint can be found in one of our affiliation's working paper repository: [https://www2.ingenio.upv.es/es/working-papers/personality-and-affects-researchers-emotional-intelligence](https://urldefense.com/v3/__https:/www2.ingenio.upv.es/es/working-papers/personality-and-affects-researchers-emotional-intelligence__;!!D9dNQwwGXtA!U30fcoaDEH2rdboyNKiMbIF3z_XqAMtd7GioIokmddrM0o1DPXAhSRmJROJIeTuwMB5RH1grUPGBnLPoNmD-$) . The submission introduces a major change of methodology, from regression analysis to structural equation modelling, with consequent adaptations throughout the text. The change is so substantial that the submission includes an additional author, Anabel Fernández-Mesa, expert in structural equation modelling." Please clarify whether this [conference proceeding or publication] was peer-reviewed and formally published. If this work was previously peer-reviewed and published, in the cover letter please provide the reason that this work does not constitute dual publication and should be included in the current manuscript.

Response: The preprint with the previous methodology is in a university repository, without having been peer-reviewed or formally published. In addition, the results of the study submitted to PLOS ONE are different, more in-depth and useful, due to the change in the methodology.

***Comment #5***

We note that you have indicated that data from this study are available upon request. PLOS only allows data to be available upon request if there are legal or ethical restrictions on sharing data publicly. For more information on unacceptable data access restrictions, please see [http://journals.plos.org/plosone/s/data-availability#loc-unacceptable-data-access-restrictions](https://urldefense.com/v3/__http:/journals.plos.org/plosone/s/data-availability*loc-unacceptable-data-access-restrictions__;Iw!!D9dNQwwGXtA!U30fcoaDEH2rdboyNKiMbIF3z_XqAMtd7GioIokmddrM0o1DPXAhSRmJROJIeTuwMB5RH1grUPGBnDb2BJhY$).

In your revised cover letter, please address the following prompts:

   a) If there are ethical or legal restrictions on sharing a de-identified data set, please explain them in detail (e.g., data contain potentially sensitive information, data are owned by a third-party organization, etc.) and who has imposed them (e.g., an ethics committee). Please also provide contact information for a data access committee, ethics committee, or other institutional body to which data requests may be sent.

   b) If there are no restrictions, please upload the minimal anonymized data set necessary to replicate your study findings as either Supporting Information files or to a stable, public repository and provide us with the relevant URLs, DOIs, or accession numbers. For a list of acceptable repositories, please see [http://journals.plos.org/plosone/s/data-availability#loc-recommended-repositories](https://urldefense.com/v3/__http:/journals.plos.org/plosone/s/data-availability*loc-recommended-repositories__;Iw!!D9dNQwwGXtA!U30fcoaDEH2rdboyNKiMbIF3z_XqAMtd7GioIokmddrM0o1DPXAhSRmJROJIeTuwMB5RH1grUPGBnKsJetik$).

We will update your Data Availability statement on your behalf to reflect the information you provide.

Response: Thank you for reaching this out. We consulted the Ethic Committees that approved our research. According to them, since, in order to get our informed consent, we promised respondents that the data would not be public even if anonymised, it would not be ethical to deposit the information in public repositories.

***Comment #6***

Your work is of great potential, but reviewers found some loopholes which can be improved to highlight your findings further.
Reviewer noticed that your work lacks in support of arguments. I suggest you cite and compare your work findings to recently published work on big five personality traits to reflect better and explain your findings. You can consider articles such as,
[https://www.tandfonline.com/doi/abs/10.1080/10447318.2023.2201551](https://urldefense.com/v3/__https:/www.tandfonline.com/doi/abs/10.1080/10447318.2023.2201551__;!!D9dNQwwGXtA!U30fcoaDEH2rdboyNKiMbIF3z_XqAMtd7GioIokmddrM0o1DPXAhSRmJROJIeTuwMB5RH1grUPGBnJrhbjcf$)
[https://doi.org/10.3389/fpsyg.2022.956281](https://urldefense.com/v3/__https:/doi.org/10.3389/fpsyg.2022.956281__;!!D9dNQwwGXtA!U30fcoaDEH2rdboyNKiMbIF3z_XqAMtd7GioIokmddrM0o1DPXAhSRmJROJIeTuwMB5RH1grUPGBnB5B7sEP$)

Response: We have improved the introduction by discussing some articles like the ones you indicate in order to enrich the argumentation.

We hope to have fully addressed all your concerns and the concerns by the two anonymous reviewers in this new version of the manuscript.

Best regards,

The authors

**TO THE ATTENTION OF REVIEWER 1**

***General comment***

The article is indeed very interesting and thoroughly performed, but in my opinion the authors should focus better on outlining the importance of their study instead of describing the review of the literature since this is a research article. Therefore, please remove the unnecessary details from the first section and mention only an introduction regarding the current study.

**Response.** Thank you very much for reading our paper and giving us detailed advice about how to improve it. We really appreciate this opportunity. We hope to have properly addressed your concerns. Concretely, in this new version of the manuscript we have highlighted the importance of the study removing the unnecessary details from the first section and adjusting the introduction.

**TO THE ATTENTION OF REVIEWER 2**

***General comment***

Thank you for the opportunity to review the manuscript “Personality and emotional intelligence of researchers: the importance of affects”.

This study analysis the relationships between personality traits, emotional intelligence (EI) and positive and negative affect among the scientific population.

I make the following recommendations to improve the manuscript.

**Response.** Thank you very much for your time and effort. Your comments were very clear and detailed, facilitating us the inclusion of your suggestions. We really appreciate your constructive tone and advise.

***Comment #1***

ABSTRACT: The abstract should be concise and informative. It should briefly describe the purpose of the work, techniques and methods used, major findings with important data and conclusions. Please edit the aim of the research. Briefly mention the main aim of the work and then write the specific aim.

**Response.** Thank you. We have included your recommendations in the abstract.

***Comment #2***

Cite references in brackets (for example, “[1]” or “[2-5]” or “[3,7,9]”).

**Response.** We really appreciate this comment and have proceeded accordingly.

***Comment #3a***

INTRODUCTION: Please improve introduction.

a. Introduction should be shorter and has the most important informations. Please explain why this research is so important and why you want to fill the gaps about the personality and emotional intelligence of researchers.

**Response.** Thank you for your feedback. We have re-written this section accordingly.

***Comment #3b***

INTRODUCTION: b. Why you made so many subsections?

**Response.** Thank you for noticing this. Our introduction is made with four paragraphs. The first one is an introduction to the field, the second and third explain what has been said in the literature about it and presents the gap and the final paragraph explains how we have covered the gap.

***Comment #3c***

INTRODUCTION: c. Please clearly define the aim of the work and the hypothesis.

**Response.** Thank you very much for your comment. We have tried to better clarify the aim of the study and the hypotheses in the Introduction and in the Literature Review section.

***Comment #4a***

MATERIAL AND METHOD: Please improve this section,

a. Participants

**Response.** We have redrafted the section in order to improve clarity and the flow of reading. We have provided additional information on the ethical committees and the representativeness of the sample.

***Comment #4b***

MATERIAL AND METHOD: b. Why did you contact only with corresponding authors for publications appearing in the Web of Science (WoS) from 2013 to 2016? Why did you choose these years?

**Response.** 2013-2016 were the years previous of the study. 2016 was the last year with reliable information and we needed to go back to relatively recent times, to avoid memory bias and abundance of obsolete email addresses. The four-year window 2013-2016 resulted into 65,000 valid e-mails, which is, to the best of our knowledge, the largest population of researchers ever addressed, so it was a good starting point. On why corresponding authors, we enlarged the explanation with citations to the literature:

“The corresponding author is usually one of the lead authors and lead contributors to the content of the manuscript [46] or is perceived as so [47]. Editors appoint corresponding authors as reviewers [48] and they are considered reliable sources of knowledge about the publication and the underlying research [49]”.

***Comment #4c***

MATERIAL AND METHOD: c. Why was you not ask socioeconomic status?

**Response.** Previous studies have not included the socioeconomic status when studying emotional intelligence [1]. This comment has made us to deeply think about the importance of socioeconomic status for emotional intelligence of the individual and consequently we have included it as an interesting future research line in the discussion section.

Reference

1. Zhou X, Sun X, Wang Z, Jiang T. Association between conscientiousness and team emotional intelligence: A moderated mediation model. Medicine (Baltimore) [Internet]. 2022;101(42): e31001. Available from: <http://dx.doi.org/10.1097/MD.0000000000031001>

***Comment #4d***

MATERIAL AND METHOD: d. Please rewrite the characteristic of the respondents.

**Response.** We have followed your suggestion and have rewritten this section (design of the study) adding the characteristics of the respondents to simplify the exposition.

***Comment #4e***

MATERIAL AND METHOD: e. Please add: exlusion and inclusion criteria f. It seems necessary to clarify how the representative sample was calculated and to make a flowchart of the enrollment of respondents and study procedures. You should explain the sample size calculation in order to know if this sample is representative.

**Response.** Thank you for noticing this. We have redrafted the section to clarify exclusion and inclusion criteria, and the enrolment of respondents. We have included the following sentence: “7,463 gave valid answers to all of the questionnaires analysed, that is, a response rate of 11.48 % and a sample size with a 95% confidence level and a 1% margin of error, which is representative of the population”.

***Comment #4g***

MATERIAL AND METHOD: g. Statistical Analysis: please improve this section. Did you use only Pearson correlation? The main issue of the paper is that, the description of the statistical analysis plan is unclear. While there are many results that are presented, only one paragraph was devoted to the statistical analysis. The authors need to pay more attention into the details of analysis plan in order to make the paper scientifically rigorous. Please add all information about analysis in this section, not in the results.

**Response.** You are totally right that the whole section was unclear. We have structured the information in a different way. The journal specifies that a section of “materials and methods” (called in this way) must be included. This section starts now with “design of the study” with a detailed information of the procedure of gathering the data. Then “measures” in which we explain how we measure the different concepts (all scales have been taken from previous literature). And finally, results in which we have included: first, the validation of the scales (psychometric properties) and second, the results of the structural equation modelling (assessing the structural model).

***Comment #4h***

MATERIAL AND METHOD: h. The design of the study is not explained, please describe it.

**Response.** Thank you, we have included a section on the study design.

***Comment #4i***

MATERIAL AND METHOD: i. I think you should write a section related to Ethical Considerations. You should clarify the ethics of your research. Please add sentence: „The study was conducted according to the guidelines of the Declaration of Helsinki and approved by the Ethics Committee of the …”.

**Response.** You are totally right. In fact, we passed two ethical evaluations of the study before conducting it and we included all the feedback in the performance of our study in order to reach proficiency in Ethical procedure. Thanks to your comment we have inserted a sentence along these lines, including a mention to the granting ethical committees:

“The project received a favourable report from the Ethics Committees of the Spanish National Research Council (CSIC) and of the Polytechnic University of Valencia.”

***Comment #4j***

RESULTS: i. each of the results should be mentioned in more depth and more **readable** for the reader.

**Response.** Thank you for noticing this. We have re-written this section to make it more accessible to the reader.

***Comment #4k***

DISCUSSION: Please improve this section.

i. The discussion of the results should be done in a more in-depth way.

ii. The discussion should discuss the implications of the findings in general and be supported by relevant and related articles.

iii. I miss an introductory paragraph in which the aim of the research and the main results found are remembered. In general, the wording of the discussion could be better. I suggest preparing some kind of summary with gaps indication and future perspective

**Response.** Thank you for your detailed feedback. We have followed your suggestions and proceeded accordingly.

***Comment #4l***

CONCLUSION: The conclusion must include a summary of the key ideas and the most important issues raised throughout the research paper. Please add this subsection!

**Response.** Thank you very much for your suggestion, we have added a section with the conclusions of the study.

***Comment #4m***

LIMITATION: please add limitations like subsection. Please discuss widely the advantages and disadvantages of this research, identify knowledge gaps in the existing literature, and try to indicate the needs for future research.

**Response.** Thank you very much for your comment, we have added the limitations in a section with the same name and included your suggestions.

Thank you for your constructive and helpful comments, which, in our view, helped improve the paper. We would be more than happy to make further changes/respond to any additional concerns that you may have.
