# Supplementary material for: Personality and emotional intelligence of researchers: The importance of affects
Source: PLoS One. 2024 Aug 9;19(8):e0304905. doi: 10.1371/journal.pone.0304905 (PMC11315323; doi:10.1371/journal.pone.0304905)
Supplement: Attachment — Submitted filename: Response_to_reviewers.docx [file pone.0304905.s002.docx]

March 6th, 2024

Dear Professor Sohaib Mustafa,

Thank you for your invitation to revise and resubmit our paper in this second round of revision and for giving us detailed instructions about how to perform the revision.

We appreciate all the comments from reviewers 3 and 4, they have provided valuable feedback which has helped us to improve our paper.

We hope to have fully addressed all your concerns and the concerns by the two anonymous reviewers from the first round and from reviewers 3 and 4 from the second round in this new version of the manuscript.

Best regards,

The authors

**TO THE ATTENTION OF REVIEWER 3**

***General comment***

This manuscript delves into the crucial role that the Big Five personality traits play in the emotional intelligence of researchers. The subject matter of this study is intriguing, and a substantial dataset has been employed to validate the article’s hypothesis. However, the reviewers have identified several areas for improvement.

**Response.** Thank you very much for reading our paper and giving us detailed advice about how to improve it. We really appreciate this opportunity. We hope to have properly addressed your concerns. Concretely, in this new version of the manuscript we have updated the introduction section, the literature review and hypotheses, included some paragraphs on the concept of personality traits and pro-environmental behaviour and technological innovation (these last two topic as a promising future research). We have changed the use of pronouns we and I, as well as subjective expressions like “may be”. And finally, we have also included more detail of the design of the study.

***Comment #1***

The introduction of the manuscript could benefit from a more thorough explanation. This should underscore the research question posed by the article, the objectives of the research, and the significance of the study.

**Response.** Thank you for the recommendation. We have rewritten and reorganised the introduction accordingly, trying to make it more comprehensive, making the problem posed and the proposed objectives more evident.

***Comment #2***

The article would be strengthened by a more exhaustive review of the relevant literature, which would help to substantiate the hypotheses presented.

**Response.** We appreciate your suggestion. We have reviewed the literature again and tried to restructure and improve the literature review section as proposed.

***Comment #3***

Given that Plos One is a multidisciplinary journal with a diverse readership, it would be beneficial for the author to dedicate a few paragraphs to introducing the concept of personality traits. Additionally, recent comprehensive discussions on technological innovation and pro-environmental behavior should be included. This would enable readers to better comprehend the importance of this study.

**Response.** Thank you very much for bringing this issue to our attention. The information on personality traits has been expanded in the literature review section, and their relationship to technological innovation and pro-environmental behaviour has also been included as a possibility for future study in the relevant section.

***Comment #4***

The manuscript’s academic writing style requires enhancement The use of subjective pronouns such as “we” and “I”, as well as subjective expressions like “may be”, should be avoided to maintain a professional tone throughout the article.

**Response.** We are very grateful for your comment. We have examined the writing style throughout the article and tried to improve the tone as much as possible by applying your suggestions. We have also hired an English reviewer to edit the whole manuscript.

***Comment #5***

In the “design of the study” section, the author should provide a more detailed account of the data collection process. This could include information about the platform used to distribute the questionnaire. It would also be helpful to report some basic demographic characteristics of the respondents, such as their tenure in the research industry and their roles within it (e.g., graduate student, junior scholar, senior scholar, manager, etc.).

**Response.** The platform used was Qualtrics. We prefer not to disclose it in the paper to avoid promotion of a private business firm who is already charging a license fee to our organisation for the use of its proprietary software.

We included demographic characteristics regarding organisation type and tenure. We included a self-citation to a report with the full demographics –anonymised for the revision process.

Thank you for your valuable and beneficial feedback, which we believe contributed to enhancing the paper. We are willing and eager to implement additional revisions or address any further concerns you may have.

**TO THE ATTENTION OF REVIEWER 4**

***General comment***

It’s an interesting study however authors are needed to incorporate certain changes to make this study publishable.

**Response**. Thank you very much for your time and effort. Your comments were very clear and detailed, facilitating us the inclusion of your suggestions. We really appreciate your constructive tone and advise.

***Comment #1***

The introduction section of the study needs improvement. The research background (common ground) and gap (complexity) are unclearly covered. Please allow me to offer an alternative and in my opinion, more intuitive and attractive way of structuring an introduction. I suggest that the authors follow the suggestions of Lange and Pfarrer, (2017) when structuring the introduction. Such structure allows completing the introduction with five major paragraphs with central story line.

Lange,D., & Pfarrer, M.D. (2017). Editors' comments: Sense and structure- The core building blocks of an AMR article. Academy of Management Review, 42(3), 407-416

**Response.** Thank you for your comments and suggested literature. We have included your recommendations in the introduction by reorganising and completing the details proposed in the suggested article.

***Comment #2***

I would also suggest to cite additional relevant literature which demonstrate the affective health of knowledge workers that is crucial for the betterment of researchers' affective and practical well-being cum enhanced performance.

Shafait, Z., & Huang, J. (2023). Exploring the Nexus of Emotional Intelligence and University Performance: An Investigation Through Perceived Organizational Support and Innovative Work Behavior. Psychology Research and Behavior Management, 4295-4313.

**Response.** We are very grateful for this comment and, again, for the suggested bibliography. We have proceeded accordingly by checking for new, more recent bibliography such as the one you suggest, making the research more up to date.

***Comment #3***

Literature/ use of theory. As a rule of thumb, make the lit review about the same length as the discussion. But you cannot make this more than 1800 words long, to stay within your word limit. In terms of the content, you need to go beyond describing a series of relevant references and tell us how your interpretation of the literature shows the gaps that exist, and how the proposed approach to the literature brings about novel opportunities to reinterpret the literature that will allow advancement in our understanding in the field. The literature review is too descriptive, and I cannot see the wood for the trees.

Please work on your theoretical underpinnings. How theory can be fixed with these variables and can contribute. Incorporating the “WHY” factor is important.

**Response.** Thank you for your feedback. We have re-written this section accordingly, so that the introduction and literature is now very similar in length to the discussion, we have tried to go further with the explanations and worked on the theoretical foundations as much as possible.

***Comment #4***

Methodology. You need to better justify your choice of methodology, and whether this is either innovative or the standard approach, for your theoretical framework (bearing in mind that the theoretical framework is not clearly defined).

**Response.** Thank you for noticing this. We have included a justification of the standard quantitative approach followed and the choice of survey and structural equation modelling methods.

***Comment #5***

Results and discussion. While the results are rigorous, their interpretation is quite descriptive and lacks originality. The format of your writing means that you are playing safe here, for example, you mechanically show how your findings corroborate aspects of the literature that were already well-known to the readers. Instead, you ought to be more ambitious here, and show what’s really new about your findings, but also how does your work expand the current understanding of what the literature has already reported, how you are saying something that makes a substantial change in our collective understanding that all authors studying this field should be aware of and cite in the future. Your contribution needs to be much more explicit; your discussion needs to genuinely expand the boundaries of knowledge.

**Response.** Thank you very much for your comment. To improve this section, it has been rewritten taking into account your suggestions, trying to show more ambitiously the implications of the results and what they really imply.

***Comment #6***

Discussion. This needs to be a coherent and cohesive set of arguments that take us beyond this study in particular and help us see the relevance of what you have found to the wider world. You need to emphasize the significance of your work: How does your finding help us transform researchers towards being more mental-health friendly?

**Response.** We have included your suggestions in the section to improve the clarity and relevance of the results in society. We have also emphasised how this helps researchers to be more mental-health friendly.

***Comment #7***

Conclusions. This section needs more than a summary of the previous sections and adds value. You need to emphasize and explicitly spell out the contribution to the theory that your analysis brings about.

Discussion should be done considering the tested hypotheses. Please read and cite the latest papers as there are a lot of recent papers on this topic on the Web of Science.

Revisit your theoretical implication. Didn’t find any theory and you have concluded your theoretical implications.

**Response.** Thank you for all your suggestions. We have tried to add value in the conclusion by adding ideas that make the contributions of our results more explicit. We have also added new, more recent citations and revised the theoretical contribution included.

Thank you for your constructive and helpful comments, which, in our view, helped improve the paper. We would be more than happy to make further changes/respond to any additional concerns that you may have.
