# Supplementary material for: Personality and emotional intelligence of researchers: The importance of affects
Source: PLoS One. 2024 Aug 9;19(8):e0304905. doi: 10.1371/journal.pone.0304905 (PMC11315323; doi:10.1371/journal.pone.0304905)
Supplement: Attachment — Submitted filename: Response_to_reviewers.docx [file pone.0304905.s003.docx]

May 9th, 2024

Dear Professor Sohaib Mustafa,

Thank you for your invitation to revise and resubmit our paper in this third round of revision and for giving us detailed instructions about how to proceed.

We appreciate the comments from reviewer 3 that has helped us to improve our paper for the last version and thank you reviewer 4 for his/her suggestion of the acceptance of the paper. It has been a pleasure to work under both guidance.

We hope to have fully addressed the concern by reviewer 3 in this last version of the manuscript.

Best regards,

The authors

**TO THE ATTENTION OF REVIEWER 3**

***General comment***

The reviewers believe that the manuscript has been revised and improved significantly by the authors.

**Response.** We are glad to know that you have notice the improvement. Thank you to help us to bring the paper to a higher level of quality.

***Comment #1***

Before recommending acceptance of the manuscript, the reviewer also has a small suggestion for the authors to consider: the manuscript will further highlight the need for the research question, especially whether the group of academic workers is representative and whether the findings of this study have chance. Be extended to more groups and have possible contributions to application-based applications.

**Response.** You are totally right. We have better clarified the gap and aim of the study in the introduction.

P. 3 (introduction):

*“At the core of managing these emotional complexities lies emotional intelligence (EI), essential for regulating one´s emotions and navigating challenging situations (1). In particular, researchers must constantly manage intense feelings of excitement, frustration, and self-doubt as they pursue discoveries, secure funding, and face the peer review process. Possessing high EI can help researchers maintain focus, foster productive collaborations, and persevere through the setbacks inherent in scientific research [5].*

*Despite the importance of EI in research settings, our understanding of researchers' emotional intelligence remains limited [3], failing to reflect the societal significance of these knowledge generators. Researchers often exhibit unique personality traits, such as introversion and neuroticism [6, 7], which may pose challenges in developing and utilizing emotional intelligence. Addressing this gap in the literature is essential, as enhancing researchers' EI could lead to improved well-being, increased research productivity, and more impactful scientific contributions.”*

Additionally, we have clarified the wide representation of researchers from every discipline in the methods (p. 11):

*The distribution by science field is as follows: technological sciences (14%), medical sciences (24%), life sciences (16%), natural sciences (24.48%) and social sciences (21%). This broad representation of researchers underscores the diversity of participants in the study.*

The sample includes researchers that have published WoS papers (working is universities, research organisations, health establishments, business firms, public administrations and non-governmental organisations). But the results could apply to researchers that do not publish WoS papers. So, in the conclusions we have highlighted the representativeness of our sample and clarified that the results could also be extended to other groups.

P. 31 and 32:

*“From a practical perspective, these confirmations contribute with information for designing programmes aimed at fostering scientific excellence. The extensive sample size of this study enables the findings to be applicable various settings, including universities, research organisations, healthcare facilities, businesses and other organisations across a wide range of scientific fields (technological, medical, life, natural and social sciences). The findings are highly likely to be relevant to researchers who were not included in our sample, as they experience comparable pressures. This includes researchers in countries, languages or science fields that are underrepresented in the WoS, as well as those working in organisations where publishing holds less significance, such as businesses or non-governmental organizations.*

*These results offer insights into the importance of understanding and managing emotions, as well as the beneficial role of positive affects. Implementing strategies to promote positive affects and mitigate negative ones is crucial. Researchers often encounter frustrating situations such as the rejection of their work by the scientific community despite their significant efforts. This inability to publish their findings can be emotionally burdensome. Therefore, in addition to considering the influence of the big five personality traits, it is recommended to take into account EI and positive affects when addressing the emotional challenges associated with such setbacks.”*

Thank you for your insightful and helpful input, which we believe has helped improve the paper. We hope you will find the article ready for publication, but if you consider that it still needs any adjustment, we are eager to implement any additional concern you may have.

**TO THE ATTENTION OF REVIEWER 4**

***General comment***

Authors have responded to the raised concerns comprehensively; therefore, I suggest acceptance of this manuscript in current form.

**Response**. Thank you sincerely for granting us the chance to enhance the paper and aiding us in crafting a revised edition of the manuscript. We are deeply grateful for the opportunity you've given us to publish the article.
